# Ransomware Attack Associated With Disruptions at Adjacent Emergency Departments in the US

**DOI:** 10.1001/jamanetworkopen.2023.12270

**Published:** 2023-05-08

**Authors:** Christian Dameff, Jeffrey Tully, Theodore C. Chan, Edward M. Castillo, Stefan Savage, Patricia Maysent, Thomas M. Hemmen, Brian J. Clay, Christopher A. Longhurst

**Affiliations:** 1 Department of Emergency Medicine, University of California, San Diego; 2 Department of Biomedical Informatics, University of California, San Diego; 3 Department of Computer Science and Engineering, University of California, San Diego; 4 Department of Anesthesiology, University of California, San Diego; 5 Office of the University of California, San Diego Health Chief Executive Officer, University of California, San Diego; 6 Department of Neurosciences, University of California, San Diego

## Abstract

**Importance:**

Cyberattacks on health care delivery organizations are increasing in frequency and sophistication. Ransomware infections have been associated with significant operational disruption, but data describing regional associations of these cyberattacks with neighboring hospitals have not been previously reported, to our knowledge.

**Objective:**

To examine an institution’s emergency department (ED) patient volume and stroke care metrics during a month-long ransomware attack on a geographically proximal but separate health care delivery organization.

**Design, Setting, and Participants:**

This before and after cohort study compares adult and pediatric patient volume and stroke care metrics of 2 US urban academic EDs in the 4 weeks prior to the ransomware attack on May 1, 2021 (April 3-30, 2021), as well as during the attack and recovery (May 1-28, 2021) and 4 weeks after the attack and recovery (May 29 to June 25, 2021). The 2 EDs had a combined mean annual census of more than 70 000 care encounters and 11% of San Diego County’s total acute inpatient discharges. The health care delivery organization targeted by the ransomware constitutes approximately 25% of the regional inpatient discharges.

**Exposure:**

A month-long ransomware cyberattack on 4 adjacent hospitals.

**Main Outcomes and Measures:**

Emergency department encounter volumes (census), temporal throughput, regional diversion of emergency medical services (EMS), and stroke care metrics.

**Results:**

This study evaluated 19 857 ED visits at the unaffected ED: 6114 (mean [SD] age, 49.6 [19.3] years; 2931 [47.9%] female patients; 1663 [27.2%] Hispanic, 677 [11.1%] non-Hispanic Black, and 2678 [43.8%] non-Hispanic White patients) in the preattack phase, 7039 (mean [SD] age, 49.8 [19.5] years; 3377 [48.0%] female patients; 1840 [26.1%] Hispanic, 778 [11.1%] non-Hispanic Black, and 3168 [45.0%] non-Hispanic White patients) in the attack and recovery phase, and 6704 (mean [SD] age, 48.8 [19.6] years; 3326 [49.5%] female patients; 1753 [26.1%] Hispanic, 725 [10.8%] non-Hispanic Black, and 3012 [44.9%] non-Hispanic White patients) in the postattack phase. Compared with the preattack phase, during the attack phase, there were significant associated increases in the daily mean (SD) ED census (218.4 [18.9] vs 251.4 [35.2]; *P* < .001), EMS arrivals (1741 [28.8] vs 2354 [33.7]; *P* < .001), admissions (1614 [26.4] vs 1722 [24.5]; *P* = .01), patients leaving without being seen (158 [2.6] vs 360 [5.1]; *P* < .001), and patients leaving against medical advice (107 [1.8] vs 161 [2.3]; *P* = .03). There were also significant associated increases during the attack phase compared with the preattack phase in median waiting room times (21 minutes [IQR, 7-62 minutes] vs 31 minutes [IQR, 9-89 minutes]; *P* < .001) and total ED length of stay for admitted patients (614 minutes [IQR, 424-1093 minutes] vs 822 minutes [IQR, 497-1524 minutes]; *P* < .001). There was also a significant increase in stroke code activations during the attack phase compared with the preattack phase (59 vs 102; *P* = .01) as well as confirmed strokes (22 vs 47; *P* = .02).

**Conclusions and Relevance:**

This study found that hospitals adjacent to health care delivery organizations affected by ransomware attacks may see increases in patient census and may experience resource constraints affecting time-sensitive care for conditions such as acute stroke. These findings suggest that targeted hospital cyberattacks may be associated with disruptions of health care delivery at nontargeted hospitals within a community and should be considered a regional disaster.

## Key Points

**Question:**

What are the associated regional health care disruptions in hospitals adjacent to health care systems under ransomware cyberattack?

**Findings:**

This cohort study of 2 academic urban emergency departments (EDs) adjacent to a health care delivery organization under a month-long ransomware attack evaluated 19 857 ED visits at the unaffected ED: 6114 in the preattack phase, 7039 in the attack and recovery phase, and 6704 in the postattack phase. During the attack and postattack phases, significant increases in patient census, ambulance arrivals, waiting room times, patients left without being seen, total patient length of stay, county-wide emergency medical services diversion, and acute stroke care metrics were seen in the unaffected ED.

**Meaning:**

This study suggests that health care cyberattacks such as ransomware are associated with greater disruptions to regional hospitals and should be treated as disasters, necessitating coordinated planning and response efforts.

## Teaser

This cohort study examines a hospital’s emergency department (ED) patient volume and stroke care metrics during a month-long ransomware attack on a geographically proximal but separate health care delivery organization.

## Introduction

Health care delivery organizations (HDOs) are increasingly dependent on internet-connected computer networks and medical devices for enterprise workflows and patient care. Protected health information and financial data render HDOs attractive targets for cybersecurity attacks, the consequences of which have been previously described.^1,2,3^ Although cyberattacks on HDOs continue to increase in frequency^4,5,6^ and the financial and operational effects of such incidents are documented,^7^ the literature is largely bereft of data demonstrating an adverse effect on patient care workflows or care outcomes.

A growing proportion of cyberattacks on HDOs use ransomware, a subset of malicious software known as malware.^8^ Ransomware programs are designed to infect a network and render the data and functionality contained within that system inaccessible until a monetary ransom is paid. The potential for significant ransomware-induced operational disruption was demonstrated by the WannaCry virus, which in 2017 infected more than 80 hospitals in the UK’s National Health Service, resulting in ambulance diversions, canceled surgical procedures, and delayed oncology care.^9,10,11^

Despite improved awareness^12^ and an increasing focus on enterprise cybersecurity,^13^ the incidence of ransomware attacks on HDOs has markedly increased over the past decade.^14^ During the COVID-19 pandemic, serious ransomware infections loaded additional stress on HDOs, with 1 attack in September 2020 affecting more than 200 affiliated facilities across the US, demonstrating the rapid spread and disregard for geography that can arise after a single intrusion.^15^ Attacks focusing specifically on institutions working on the development and testing of a vaccine have drawn increased concern from law enforcement and national security agencies.^16,17^

The financial effect of ransomware attacks extends beyond the initial ransom, which may itself cost millions of dollars.^4^ Prolonged disability of critical hospital infrastructure can result in more severe losses, with the September 2020 attack previously mentioned resulting in a cost of $67 million to the affected health system.^18^ To our knowledge, the only ransomware patient outcomes data published in the literature are in a retrospective impact analysis by Ghafur and colleagues^19^ of the WannaCry attack; the analysis did not find a difference in mortality between the baseline of the National Health Service facilities and the week of the attack, despite decreases in admissions and emergency department (ED) visits, although the attack was halted after less than 24 hours and the subsequent technical recovery was relatively rapid. To our knowledge, data from a ransomware attack resulting in a prolonged disruption have not been reported.

On the evening of May 1, 2021, an HDO with 4 acute care hospitals with more than 1300 combined acute inpatient beds and 19 outpatient facilities was infected with ransomware. According to local media reports, this attack resulted in acute loss of the electronic health records, imaging systems, and telemedicine capabilities.^20^ Clinicians reverted to manual processes, including the use of paper medical records, while emergency ambulance traffic was diverted to unaffected facilities.^21^ Records of nearly 150 000 patients were compromised as a result of the breach,^22^ and operational disruptions persisted for 4 weeks after the attack was first detected.^23^

We report on the operational and patient volume data at an adjacent, uninfected HDO during the period of this attack. We also report on the regional emergency medical services (EMS) diversion data and ED stroke care metrics. We examine the association of the disruption incurred by the infected HDOs with operational disruptions at other hospitals in the same regional health care ecosystem.

## Methods

This study and participant informed consent were deemed exempt from institutional review board review by the University of California, San Diego, institutional review board because patient identifiers were not collected. This study conforms to the Strengthening the Reporting of Observational Studies in Epidemiology (STROBE) reporting guideline.^24^

### Study Design

This was a retrospective before and after cohort study comparing ED metrics of 2 EDs in proximity to the HDO under attack. Data were collected for the 4 weeks prior to the attack (weeks 1-4: April 3-30, 2021), during the attack and recovery (weeks 5-8: May 1-28), and 4 weeks after full operational restoration (weeks 9-12: May 29 to June 25).

### Study Setting

San Diego County has 4 large health care systems (HDO A, B, C, and D), which account for approximately 73% of the inpatient discharges in the region. The characteristics of these systems are presented in the eTable in Supplement 1. The remainder of the market share is distributed among 2 smaller HDOs and a large regional children’s hospital.^25^

All 4 hospitals (comprising acute care facilities for HDO A) infected with ransomware were located in San Diego County, and when combined, they represent approximately 25% of all acute inpatient discharges for the region.^25^ Three hospitals are designated stroke receiving centers, and 1 hospital is a comprehensive stroke and level 1 trauma center.^26^

This study reports data from HDO B, which operates 2 large hospitals with a combined 11% regional inpatient discharge rate. Both hospitals are designated stroke receiving centers, and one is a level 1 trauma center. Both hospitals have EDs with a combined yearly census of more than 70 000 patients.

The 2 remaining HDOs (C and D) maintain 6 hospitals accounting for 37% of the region’s inpatient discharges. Two hospitals are comprehensive stroke centers, and 4 are primary stroke receiving centers.

### Patient Population

Adult and pediatric patients presenting to the regional (HDO B) EDs during the study period were included and combined in this analysis. *Census* is defined as the total number of patient visits to the ED. Patients who presented multiple times to the ED during the study period had each encounter counted as a distinct visit in the ED census. Patients who left before triage or were immediately sent to another department (trauma, labor and delivery, or burn) before emergency physician evaluation were excluded. Patient demographic characteristics (eg, age, sex, and race and ethnicity [non-Hispanic White, Hispanic, non-Hispanic Black, non-Hispanic Asian or Pacific Islander, other, multiracial, or unknown]) were extracted from the electronic health records and were defined by the patients themselves. *Emergency medical services ambulance census* was defined as any basic or advanced life support ambulance patient arrival to the ED (EMS arrival).

### Disposition and Temporal Classification

*Hospital admissions* were defined as any ED patient being placed in either observation or admission status to any hospital service (eg, medicine, surgery, or psychiatry). Patients placed in ED observation were not included unless they were later admitted to a hospital service. Patients who left without being seen were defined as those who had completed nurse triage but had not yet been seen by an ED clinician (intern, resident, nurse practitioner, physician assistant, or attending physician). Patients who left against medical advice were defined as those electing to depart against the recommendations of the attending physician after evaluation. *Eloped patients* were defined as any patient who could not be located after being placed in a treatment area after nurse triage and clinician evaluation.

*Emergency department revisits* were defined as any patient presenting during the study period who returned for a subsequent ED encounter within 7 days from their prior ED discharge or transfer to an outside facility. Patients presenting to either HDO B site constituted an ED revisit. Patients transferred between EDs were not characterized as revisits. *Emergency department readmissions* were defined as any ED patient readmitted to any service within 30 days from their last hospital discharge. Emergency department revisits and readmissions were attributed to the original presentation date even if the subsequent visit or readmission occurred during a different attack phase (eg, patient was seen during week 4 but revisited during week 5).

*Length of stay* (LOS) was defined as the total time in minutes from patient arrival to their physical departure from the ED (admitted or discharged). *Door-to-room time* was defined as the time from ED arrival to placement in an ED treatment area and approximates “waiting room times.”

### Diversion

The county of San Diego EMS, a division of San Diego County Fire, records and sums on a daily basis the cumulative number of minutes that each hospital in San Diego County is on ED EMS diversion. *Diversion* is a temporary elective designation an individual hospital may trigger to redirect ambulance traffic away from their ED to another. At the time of the attack, diversion status necessity was reevaluated every 2 hours. Hospitals can also go on diversion during declared internal disasters, which HDO B initiated during the start of the ransomware attack, which did not require bihourly reevaluation.

### Stroke Classification

*Emergency department stroke codes* are defined as those activated in the ED by emergency physicians or by paramedics in the field, and they can include confirmed or suspected strokes. Emergency department stroke codes use the internal paging system for notification and are documented in the electronic health records as an order and are recorded in a flow sheet. *Stroke *was defined as any ED patient discharged with a diagnosis of ischemic stroke, intracranial hemorrhage, subarachnoid hemorrhage, or transient ischemic attack per *International Statistical Classification of Diseases and Related Health Problems, Tenth Revision* codes as defined by the American Heart Association Get With the Guidelines program.^27^ *Receipt of acute stroke treatment* was defined as any patient receiving thrombolytics, such as tissue plasminogen activator (tPA) or endovascular treatment. *Door–to–computed tomography (CT) scanner time* was defined as the time from ED presentation to CT scan completion in minutes. *Door-to-tPA time* was defined as the time from ED presentation to initiation of tPA infusion for stroke codes in minutes. *Door-to-groin puncture time* was defined as the time from ED presentation to confirmed procedural vascular access for endovascular treatment. Cases were identified through our institution’s internal stroke registry.

### Data Collection

Census, recurrence, throughput, and stroke data were extracted from the electronic health records using structured query language using the Clarity database (Epic). Diversion data were provided by San Diego County EMS as part of routine internal data collection efforts. Extracted data were stored on an internal secured server.

### Statistical Analysis

Data were grouped into the 3 study periods. Descriptive analysis for demographic characteristics and census, recurrence, throughput, and performance measures were conducted for each group. Mean (SD) values or median (IQR) values are reported for continuous data as appropriate, and frequencies and percentages are reported for categorical data. Comparisons are presented overall and between each group when significant. For the overall comparisons, a 1-way analysis of variance was performed for continuous normally distributed data, and the Kruskal-Wallis test was performed for nonparametric data. Comparisons between groups of significant measures were completed using the *t* test or the Mann-Whitney test as appropriate. The χ^2^ test was used for all categorical data. All *P* values were from 2-sided tests and results were deemed statistically significant at *P* < .05. Because this cohort study has an observational study design assessing plausible associations with measures between groups, a *P* value correction was not used. Rather, because these comparisons were performed to identify differences in each measure over the 3 phases, the importance of these differences was also interpreted based on their association with clinical operations. A brief assessment of the association of a correction with clinical operations resulted in a minimal association with the interpretation of data. Statistical analysis was performed using IBM SPSS Statistics for Windows, version 27 (IBM Corp).

## Results

During the study period, we evaluated 19 857 ED visits: 6114 (mean [SD] age, 49.6 [19.3] years; 2931 [47.9%] female patients; 1663 [27.2%] Hispanic, 677 [11.1%] non-Hispanic Black, and 2678 [43.8%] non-Hispanic White patients) in the preattack phase, 7039 (mean [SD] age, 49.8 [19.5] years; 3377 [48.0%] female patients; 1840 [26.1%] Hispanic, 778 [11.1%] non-Hispanic Black, and 3168 [45.0%] non-Hispanic White patients) in the attack and recovery phase, and 6704 (mean [SD] age, 48.8 [19.6] years; 3326 [49.5%] female patients; 1753 [26.1%] Hispanic, 725 [10.8%] non-Hispanic Black, and 3012 [44.9%] non-Hispanic White patients) in the postattack phase. Patient demographic characteristics, including age, sex, and race and ethnicity, were similar between attack phase groups and are presented in Table 1.

**Table 1.** Patient Demographic Characteristics by Attack Phase

Variable	Patients, No (%)			*P* value
	Before attack (n = 6114)	During attack and recovery (n = 7039)	After attack (n = 6704)	
Age, mean (SD), y	49.6 (19.3)	49.8 (19.5)	48.8 (19.6)	.009
Sex				
Female	2931 (47.9)	3377 (48.0)	3316 (49.5)	.14
Male	3182 (52.0)	3657 (52.0)	3387 (50.5)	
Race and ethnicity				
Hispanic	1663 (27.2)	1840 (26.1)	1753 (26.1)	.35
Non-Hispanic Asian or Pacific Islander	388 (6.3)	423 (6.0)	463 (6.9)	
Non-Hispanic Black	677 (11.1)	778 (11.1)	725 (10.8)	
Non-Hispanic White	2678 (43.8)	3168 (45.0)	3012 (44.9)	
Other, mixed race, or unknown^a^	708 (11.6)	836 (11.9)	751 (11.2)	

^a^ These data were collected in the electronic health record and are patient entered. There is an option when self-identifying race and ethnicity to select “Other” and “Mixed Race.” There is no further detail required from the patient when they self-identify.

### Census and Recurrence

**Figure 1.**
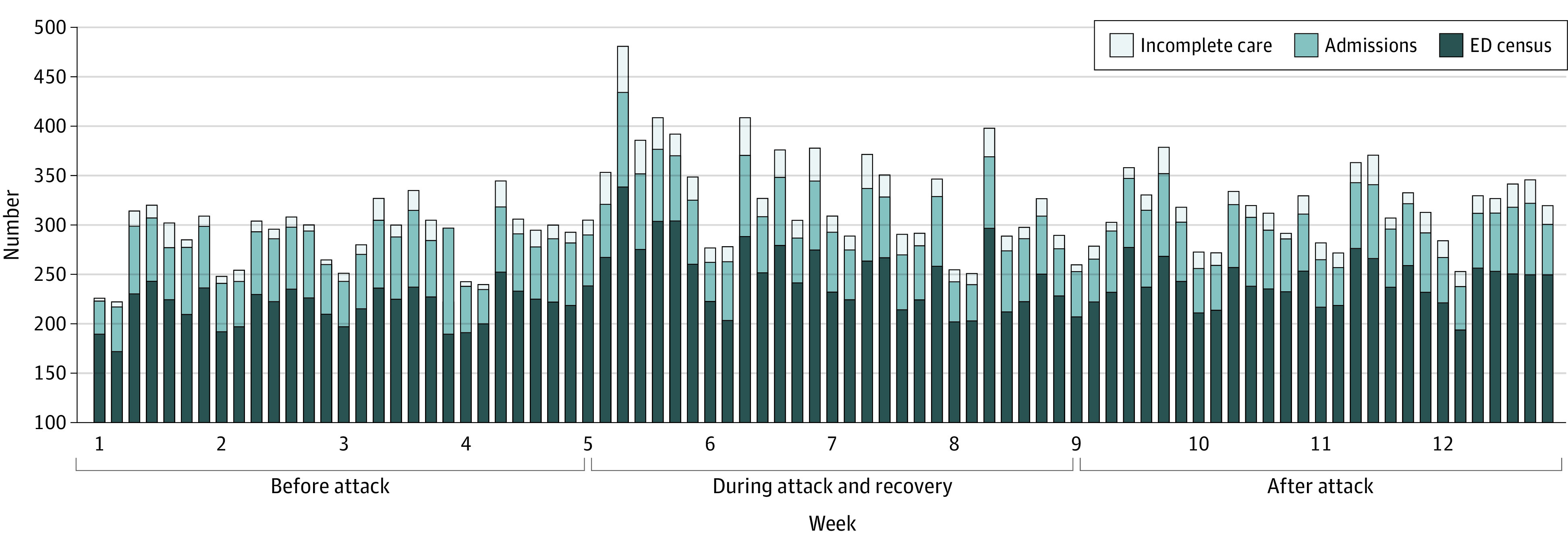
Emergency Department (ED) Census, Admissions, and Incomplete Care Per Day Incomplete care includes patients who left without being seen, patients who left against medical advice, and patients who “eloped” (could not be located after being placed in a treatment area after nurse triage and clinician evaluation).

Combined HDO B ED census and disposition metrics for the 3 study periods are presented in Table 2 and Figure 1. There was a significant increase in mean (SD) daily ED volume (ie, census) when comparing the preattack (218.4 [18.9]), attack (251.4 [35.2]), and postattack phases (239.4 [21.3]) (*P* < .001). There was a significant increase in mean (SD) EMS arrivals comparing the preattack with the attack phase (1741 [28.8] vs 2354 [33.7]; *P* < .001). There was no significant difference in EMS arrivals between the preattack and postattack phases (1741 [28.8] vs 1920 [28.9]; *P* = .90). The mean (SD) numbers of admissions were also significantly increased between the preattack and attack phases (1614 [26.4] vs 1722 [24.5]; *P* = .01, but they were not significant between the attack and postattack phases (1722 [24.5] vs 1648 [24.6]; *P* = .87). There were significant differences in the mean (SD) number of patients who left without being seen among all phases: preattack (158 [2.6]), attack (360 [5.1]), and postattack (260 [3.9]) (*P* < .001). For patients who left against medical advice, there was a significant difference between the preattack and attack phases (mean [SD], 107 [1.8] vs 161 [2.3]; *P* = .03) and no significant difference between the attack and postattack phases (161 [2.3] vs 101 [1.5]; *P* = .28). There were no significant differences among ED visits in which patients eloped, revisited within 7 days, or were readmitted within 30 days of their ED visit.

**Table 2.** Health Care Delivery Organization B ED Census, Recurrence, and Throughput Metrics

Characteristic	Before attack (n = 6114)	During attack and recovery (n = 7039)	After attack (n = 6704)	*P* value			
				Overall	Before attack vs attack	Attack vs after attack	Before vs after attack
ED daily census, mean (SD)	218.4 (18.9)	251.4 (35.2)	239.4 (21.3)	<.001	<.001	.13	<.001
EMS arrivals, mean (SD)	1741 (28.8)	2354 (33.7)	1920 (28.9)	<.001	<.001	<.001	.90
Admissions, mean (SD)	1614 (26.4)	1722 (24.5)	1648 (24.6)	.02	.01	.87	.02
Left without being seen, mean (SD)	158 (2.6)	360 (5.1)	260 (3.9)	<.001	<.001	<.001	<.001
Left against medical advice, mean (SD)	107 (1.8)	161 (2.3)	101 (1.5)	.002	.03	<.001	.28
Eloped, mean (SD)	77 (1.3)	102 (1.4)	91 (1.4)	.65	No further statistical analysis performed^a^		
7-d ED revisits, mean (SD)	514 (12.6)	563 (12.2)	548 (12.1)	.79	No further statistical analysis performed^a^		
30-d Readmission, mean (SD)	237 (14.7)	241 (14.0)	234 (14.2)	.85	No further statistical analysis performed^a^		
Median door-to-room time (IQR), min	21 (7-62)	31 (9-89)	23 (8-66)	<.001	<.001	<.001	.001
Admitted median total length of stay (IQR), min	614 (424-1093)	822 (497-1524)	680 (452-1271)	<.001	<.001	<.001	<.001
Discharged median total length of stay (IQR), min	290 (198-421)	307 (203-453)	298 (201-432)	<.001	<.001	.04	.03

Abbreviations: ED, emergency department; EMS, emergency medical services.

^a^ Due to insignificant overall *P* value.

### Throughput

Combined HDO B ED throughput metrics for the 4 weeks before, during, and after the cyberattack are presented in Table 2. The median ED door-to-room time (waiting room times) for the 4 weeks prior to the attack was 21 minutes (IQR, 7-62 minutes), 31 minutes (IQR, 9-89 minutes) during the 4 weeks of the attack, and 23 minutes (IQR, 8-66 minutes) for the 4 weeks after the attack. The median total LOS for admitted patients was 614 minutes (IQR, 424-1093 minutes) prior to the attack, which increased to 822 minutes (IQR, 497-1524 minutes) during the attack and decreased to 680 minutes (IQR, 452-1271 minutes) after the attack. The median total LOS for discharged patients was 290 minutes (IQR, 198-421 minutes) prior to the attack, 307 minutes (IQR, 203-453 minutes) during the attack, and 298 minutes (IQR, 201-432 minutes) after the attack. There were significant differences in all throughput metrics when compared at each phase (*P* < .001).

### Diversion

**Figure 2.**
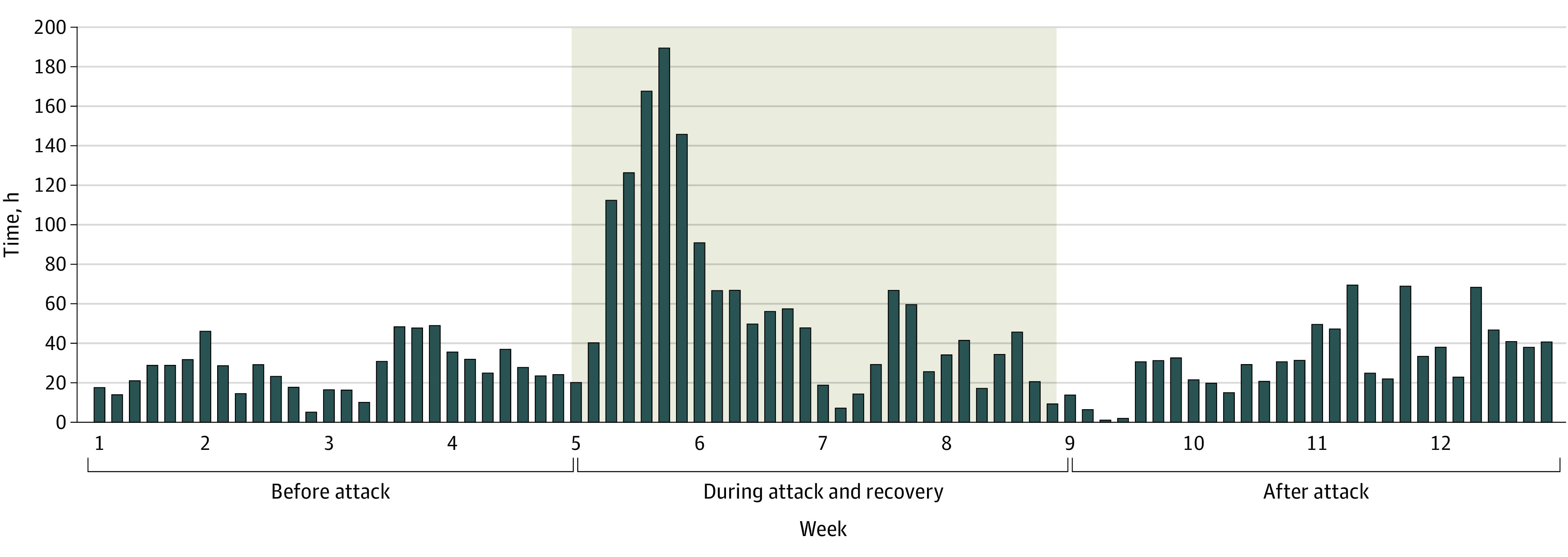
Cumulative San Diego County Emergency Medical Services Diversion Hours Per Day

San Diego County EMS reported on diversion of ambulance traffic to HDO B. They experienced a median of 27 cumulative hours (IQR, 18-32 hours) of diversion per day in the 4 weeks prior to the attack, 47 cumulative hours (IQR, 22-67 hours) per day during the 4 weeks of the attack, and 31 cumulative hours (IQR, 22-41 hours) per day of diversion in the 4 weeks after (Figure 2).

### Stroke

Combined HDO B ED stroke metrics are presented in Table 3. There was a significant increase in ED stroke code activations in the preattack phase (59), during the attack phase (103), and the postattack phase (65) (*P* = .009). There was also a significant increase in the number of confirmed strokes diagnosed when comparing the preattack phase (22), attack phase (47), and postattack phase (28) (*P* = .02). We saw increases in stroke code alerts, stroke diagnoses, and acute treatments with tPA and endovascular treatments during the cyberattack and recovery. The increased stroke alerts were not correlated with longer times to stroke imaging (CT scan), tPA administration, or time to groin puncture for endovascular treatment.

**Table 3.** Stroke Census and Performance Metrics

Characteristic	No.			*P* value			
	Before attack	During attack and recovery	After attack	Overall	Before attack vs attack	Attack vs after attack	Before vs after attack
ED stroke codes	59	103	65	.009	.01	.01	.98
Confirmed strokes	22	47	28	.02	.02	.047	.60
Acute treatment							
tPA	5	9	1	No statistical analysis performed^a^			
EVT	2	7	3	No statistical analysis performed^a^			
Total	7	16	4	No statistical analysis performed^a^			
Door–to–CT scan time, median (IQR), min	19 (11-33)	18 (9-34)	20 (10-32)	.69	No further statistical analysis performed^b^		
Door–to–tPA administration time, median (IQR), min	35 (31-87)	33 (27-44)	29^a^	No statistical analysis performed^a^			
Door–to–EVT groin puncture, median (IQR), min	85^a^	79 (59-106)	84 (81-86)	No statistical analysis performed^a^			

Abbreviations: CT, computed tomography; ED, emergency department; EVT, endovascular treatment; tPA, tissue plasminogen activator.

^a^ No statistical analysis performed due to low numbers.

^b^ Due to insignificant overall *P* value.

## Discussion

In this study, when comparing the preattack period with the attack period, there was an associated 15.1% increase in daily mean (SD) ED volume (ie, census) (from 218.4 [18.9] to 251.4 [35.2]), a 35.2% increase in mean (SD) ambulance arrivals (from 1741 [28.8] to 2354 [33.7]), a 6.7% increase in mean (SD) admissions (from 1614 [26.4] to 1722 [24.5]), a 127.8% increase in visits where patients left without being seen (from 158 [2.6] to 360 [5.1]), a 50.4% increase in visits where patients left against medical advice (from 107 [1.8] to 161 [2.3]), a 47.6% increase in median waiting room times (from 21 minutes [IQR, 7-62 minutes] to 31 minutes [IQR, 9-89 minutes]), a 33.9% increase in median total LOS for admitted patients (from 614 minutes [IQR, 424-1093 minutes] to 822 minutes [IQR, 497-1524 minutes]), and a 5.9% increase in median total LOS for discharged patients (from 290 minutes [IQR, 198-421 minutes] to 307 minutes [IQR, 203-453 minutes]) at 2 normally functioning health care facilities adjacent to 4 hospitals under ransomware attack. In the postattack phase, only EMS arrivals, patients who left against medical advice, ED stroke code activations, and confirmed strokes returned to preattack rates. Anecdotal reports from clinicians stated that the associated disruptions of the cyberattack were most pronounced during the first 2 weeks of the attack and likely the result of early chaos when no mitigations existed yet for hospitals to develop ad hoc workarounds. In the greater San Diego County area, a 74.1% increase in median total daily ED diversion time (hours) was observed when comparing the preattack period with the attack period (from 27 cumulative hours [IQR, 18-32 hours] to 47 cumulative hours [IQR, 22-67 hours]), likely associated with the increase in census and EMS arrivals at HDO B.

Acute stroke care is an example of a time-sensitive, resource-intensive, technologically dependent, and potentially lifesaving set of complex actions and decisions requiring a readily available multidisciplinary team working in close coordination. Several of the hospitals targeted by the ransomware attack are stroke centers, necessitating the transport of these high-acuity patients to a reduced number of functioning stroke centers in the region. This study showed, at HDO B, an associated 74.6% increase in stroke code activations (from 59 to 103) and 113.6% increase in confirmed strokes (from 22 to 47) from the preattack phase to the attack phase. There was no significant difference in door–to–CT scan or acute stroke treatment times. Indirect impediments to care have been associated with patient outcomes in the setting of other time-sensitive conditions, including acute myocardial infarction or cardiac arrest.^28^ It may be reasonable to consider the impact of cybersecurity disruption within such an outcomes-oriented context.

Ransomware attacks on hospital systems have previously been reported to be costly and disabling^21^; however, data about regional disruptions at neighboring hospitals, to our knowledge, have not been previously reported. Increases in health care cybersecurity incidents suggest the need for coordinated regional surge planning similar to that conducted for natural disasters.^29^ Interdisciplinary teams of technologists and clinicians are needed to address the unique challenges of cybersecurity incidents, including ransomware.^30,31,32^ These include difficulty predicting which facilities are at highest risk due to a lack of geographic association and institutional precedent, rapid spread across large distances within a hospital network affecting multiple facilities simultaneously, and protracted operational downtimes approaching weeks to months.

Hospital systems infected with ransomware can likely reduce regional effects by developing cyberattack-specific emergency operations plans to minimize recovery times in addition to engaging regional partners to proactively plan for and drill for cyberattacks. Real-time information sharing on cyber threat actors and methods can reduce the risk of spread among HDOs. Risks to specific patient populations, such as those with trauma, stroke, or myocardial infarction, should be anticipated, and measures to rapidly facilitate transfers among hospitals should be prioritized. Prolonged regional effects may necessitate consideration of reducing elective surgical cases and other extraordinary measures.

Increasing cyberattack prevention efforts and operational resiliency across all health care systems should be a high national priority.^33,34,35^ Further study on the association of cyberattacks with patient safety and quality of care is needed, although significant barriers to data collection and reporting remain given the reliance on affected electronic adverse event monitoring systems and HDO legal liability concerns.^36,37,38^

### Limitations

There are several limitations to this study, including its observational, descriptive design with no defined sample size, precluding true hypothesis testing; a relatively low incidence of stroke; regional concentration of hospitals affected by cyberattacks potentially magnifying the “spillover” effects; limited focus on emergency care, potentially missing data on the effects on outpatient and other inpatient services; and limited generalizability to outside HDOs, given large variations in geographic hospital distribution. Confounding effects, including variations in infectious diseases or trauma burden or seasonality, may be responsible for changes in ED metrics independent of the cybersecurity incident. In addition, because we did not have access to data from HDOs C and D, we were unable to examine whether similar findings occurred within those systems.

## Conclusions

This cohort study found an associated increase in ED volume (ie, census), EMS arrivals, patients who left without being seen, waiting room times, total ED LOS for admitted patients, county-wide ED diversion time, stroke code activations, and confirmed strokes at 2 hospitals near an adjacent health care organization under a month-long ransomware attack. These findings support the need for coordinated regional cyber disaster planning, further study on the potential patient care effects of cyberattacks, and continued work to build technical health care systems resilient to cyberattacks such as ransomware.

## Notes

Supplement 1. **eTable. **San Diego County Hospital Characteristics

Supplement 2.
Data Sharing Statement
