# Supplementary material for: Ransomware Attack Associated With Disruptions at Adjacent Emergency Departments in the US
Source: JAMA Netw Open. 2023 May 8;6(5):e2312270. doi: 10.1001/jamanetworkopen.2023.12270 (PMC10167570; doi:10.1001/jamanetworkopen.2023.12270)
Supplement: Supplement 1. — eTable. San Diego County Hospital Characteristics [file jamanetwopen-e2312270-s001.pdf]

Supplementary Online Content

Dameff C, Tully J, Chan TC, et al. Ransomware attack associated with disruptions at adjacent emergency departments in the US. *JAMA Netw Open.* 2023;6(5):e2312270. doi:10.1001/jamanetworkopen.2023.12270

eTable. San Diego County Hospital Characteristics

This supplementary material has been provided by the authors to give readers additional information about their work.

© 2023 Dameff C et al. *JAMA Network Open.*

eTable. San Diego County Hospital Characteristics

**Healthcare Delivery Org (HDO), Hospital**	**Acute Bed Capacity**	**Trauma Designation level**	**Stroke Receiving Center**
HDO A			
1	619	I	Y
2	432	I	Y
3	163	N/A	Y
4	173	N/A	Y
HDO B			
1	381	I	Y
2	418	N/A	Y
HDO C			
1	832	II	Y
2	443	N/A	Y
3	243	N/A	Y
4	59	N/A	Y
HDO D			
1	596	N/A	Y
2	321	N/A	Y

© 2023 Dameff C et al. *JAMA Network Open.*
