# Supplementary material for: Ransomware Attack Associated With Disruptions at Adjacent Emergency Departments in the US
Source: JAMA Netw Open. 2023 May 8;6(5):e2312270. doi: 10.1001/jamanetworkopen.2023.12270 (PMC10167570; doi:10.1001/jamanetworkopen.2023.12270)
Supplement: Supplement 2. — Data Sharing Statement [file jamanetwopen-e2312270-s002.pdf]

Data Sharing Statement

Dameff. Ransomware Attack Associated With Disruptions at Adjacent Emergency Departments in the US. *JAMA Netw Open*. Published May 08, 2023.
doi:10.1001/jamanetworkopen.2023.12270

Data
Data available: Yes
Data types: Data (not involving human participants)
How to access data: E-mail: cdameff@health.ucsd.edu
When available: With publication

Supporting Documents
Document types: None

Additional Information
Who can access the data: Researchers whose proposed use of the data has been approved
Types of analyses: For per-approved purposes.
Mechanisms of data availability: After approval of a proposal.
